# Fast and Stable Ionic Electroactive Polymer Actuators with PEDOT:PSS/(Graphene–Ag-Nanowires) Nanocomposite Electrodes

**DOI:** 10.3390/s18093126

**Published:** 2018-09-16

**Authors:** Minjeong Park, Joohee Kim, Hanjung Song, Seonpil Kim, Minhyon Jeon

**Affiliations:** 1 Department of Nanoscience and Engineering, Center for Nano Manufacturing, Inje University, Gimhae 50834, Korea; mjpark9121@gmail.com (M.P.); 21wngml@gmail.com (J.K.); hjsong@inje.ac.kr (H.S.); 2 Department of Military Information Science, Gyeongju University, Gyeongju 38065, Korea; seonpil@gu.ac.kr

**Keywords:** ionic electroactive polymer actuators, graphene, silver nanowires, poly(3,4-ethylenedioxythiophene):polystyrene sulfonate, nanocomposite electrode

## Abstract

Ionic electroactive polymer (IEAP) actuators that are driven by electrical stimuli have been widely investigated for use in practical applications. However, conventional electrodes in IEAP actuators have a serious drawback of poor durability under long-term actuation in open air, mainly because of leakage of the inner electrolyte and hydrated cations through surface cracks on the metallic electrodes. To overcome this problem, a top priority is developing new high-performance ionic polymer actuators with graphene electrodes that have superior mechanical, electrical conductivity, and electromechanical properties. However, the task is made difficultby issues such as the low electrical conductivity of graphene (G). The percolation network of silver nanowires (Ag-NWs) is believed to enhance the conductivity of graphene, while poly(3,4-ethylenedioxythiophene):polystyrene sulfonate (PEDOT:PSS), which exhibits excellent stability under ambient conditions, is expected to improve the actuation performance of IEAP actuators. In this study, we developed a very fast, stable, and durable IEAP actuator by employing electrodes made of a nanocomposite comprising PEDOT:PSS and graphene–Ag-NWs (P/(G–Ag)). The cost-effective P/(G–Ag) electrodes with high electrical conductivity displayed a smooth surface resulting from the PEDOT:PSS coating, which prevented oxidation of the surface upon exposure to air, and showedstrong bonding between the ionic polymer and the electrode surface. More interestingly, the proposed IEAP actuator based on the P/G–Ag electrode can be used in active biomedical devices, biomimetic robots, wearable electronics, and flexible soft electronics.

## 1. Introduction

Electroactive polymers (EAPs) have been considered for potential application in various fields such as robotics, aerospace, and bio-mimetics [1,2,3,4,5,6]. In particular, ionic EAPs (IEAPs) are polymers that allow mobility or diffusion of ions, and they respond mechanically to electrical stimulation by exhibiting significant changes in their shape and size through swelling, shrinkage, and warpage [1,2,7]. The simplest type of IEAP actuator consists of an electrolyte between two electrodes, like a sandwich [1,7,8,9,10]. IEAPs are active actuators that show large bending deformation upon the application of a low actuation voltage (1–5 V), and they offer advantages such as compliance, lightweight, low voltage operation, and capability of working in an aqueous medium [2,11,12,13,14,15].

The electrodes of an IEAP actuator must be flexible, but the adhesion of the flexible electrodes during deformation cycles is a major problem [2,16]. A typical IEAP actuator consists of a Nafion membrane with chemically plated Pt metal on both sides prepared by electroless plating [16,17]. Platinum is a widely used electrode material in IEAP actuators because of its high electrochemical stability and excellent electrical conductivity. However, IEAP actuators with Pt electrodes are very thick, have limited service lifetime under ambient conditions, and tend to have microcracks forming on the electrode surface after prolonged cyclic operation, resulting in low durability owing to damage to the electrode by water vapor [9,10,11]. To overcome these problems, scientists have carried out research studies on other electrode materials such as gold [18,19], copper [20], silver [21], palladium [22], and carbon nanotubes [23]. However, recent reports of IEAP actuators [24,25,26,27,28,29] show that most still use Pt electrodes prepared by electroless plating. Adoption of such actuators is limited because of their heavy weight. Therefore, it is essential to develop new high-performance ionic polymer actuators with carbon-based nonmetallic conductive electrodes [16] that have a lighter weight and better mechanical, electrical, and electromechanical properties than metal electrodes. 

Compared to other carbonaceous materials, graphene has high flexibility, low thickness, stretch ability, high electrical conductivity owing to its unique two-dimensional (2D) structure, and durability owing to its hydrophobic surface. Therefore, when compared to Pt electrodes, graphene electrodes are less prone to surface damage or water evaporation during long-term operation. However, graphene electrodes are still limited by low electrical conductivity. The sheet resistance (*R*_s_) of monolayered graphene that is grown by chemical vapor deposition (CVD) is about 1 kΩ/sq. [30], which is significantly higher than that of a Pt electrode. The grain boundaries in graphene are often line defects at the interfaces between two domains with different crystallographic orientations [30,31,32], and they affect the transport properties of CVD-grown graphene [30]. It has been theoretically predicted that the detrimental effect of these line defects can be eliminated by integrating CVD-grown graphene with one-dimensional (1D) metal nanowires [30,33]. 

Owing to the inherent low resistivity of silver metal and the narrow width of silver nanowires (Ag-NWs), a percolation network of Ag-NWs has low resistance [34]. Therefore, adding Ag-NWs to graphene will enhance its conductivity. However, because of the inherent irregular surface morphology of Ag-NWs, their poor adhesion can result in significant shorting, a poor fill factor, and easy detachment from substrates, which are all critical issues in the practical use of Ag-NWs in devices [34]. 

On the other hand, a poly(3,4-ethylenedioxythiophene):polystyrene sulfonate (PEDOT:PSS) film has excellent stability under ambient conditions [35], high transparency in the visible range, and high mechanical flexibility [36], and it can be readily prepared by conventional solution-processing techniques. These properties of PEDOT:PSS can be exploited to address the shortcomings of Ag-NWs, but a PEDOT:PSS film prepared from an aqueous PEDOT:PSS solution usually has poor conductivity [37], which is much lower than that of Ag-NWs. Therefore, a PEDOT:PSS film with sufficiently low thickness must be used to maintain the electrical conductivity of the graphene–Ag-NW electrode. 

In this study, we developed a new IEAP actuator based on PEDOT:PSS/(graphene–Ag-NWs) (hereafter abbreviated as P/(G–Ag)) nanocomposite electrodes. A graphene electrode with an embedded Ag-NW network has higher electrical conductivity than the bare Pt and bare graphene electrodes in traditional IEAP actuators. Water evaporation in the actuator during operation is also prevented because graphene is hydrophobic. The application of a PEDOT:PSS film to an IEAP actuator is expected to result in synergistic coupling between the thin nanostructured electrodes and the polymer, improve stability, and prevent detachment of Ag-NWs during long-term actuation. These electrodes for flexible actuators can be easily fabricated through solution processing, and the actuators will offer the advantages of inexpensive cost, short fabrication time, high electrical conductivity, high stability under the real-life operating conditions, rapid response, high durability, and high actuation performance.

## 2. Materials and Methods

### 2.1. Materials

Graphene, Ag-NWs, and PEDOT:PSS were used to fabricate the nanocomposite electrodes of the IEAP actuator. Graphene was synthesized by chemical vapor deposition (CVD) on Cu foil (purity: 99.8%, 35 μm) that was purchased from Nippon Ining, Japan. The Ag NW solution was purchased from Nanopyxis Co. Ltd., Jeonju, Korea. PEDOT:PSS 1.3 wt% was purchased from Heraeus, Germany. The Nafion membrane with the thickness of ~183 μm (N117) was purchased from Dupont, Wilmington, DE, USA, as the base polymer of the IEAP. Nitric acid (HNO_3_; ACS reagent grade, 70%), ammonium persulfate (APS), poly meta(methyl) acetate (PMMA; average *M*_w_: ~996,000 by gas-permeation chromatography, crystalline), and chlorobenzene (C_6_H_5_Cl; ACS reagent, purity: ≤99.5%) were purchased from Sigma-Aldrich Co., USA, and they were used to transfer graphene to the Nafion membrane. Lithium chloride (LiCl) was purchased from Sigma-Aldrich Co., Seoul, Korea. 1-ethyl-3-methylimidazolium trifluoromethylsulfanate (EMIM-Otf), an ionic liquid (IL), was purchased from Merck, Seoul, Korea. LiCl and IL were used in the ion-substitution process of the actuator. Deionized (DI) water (resistivity: >18 MΩ·cm) was used throughout the experiments. All of the materials, including sodium borohydride solution (NaBH_4_, 5 wt%), hydrochloric acid (HCl, 0.1 N), and ammonium hydroxide (NH_2_OH, 5 wt%), related to the electroless plating process were purchased from Sigma-Aldrich Co.

### 2.2. Fabrication of IEAP Actuator Based on Pt Electrode

We fabricated an IEAP actuator based on Pt electrodes using the well-known electroless plating process and compared it with an IEAP actuator based on the PEDOT:PSS/(graphene–Ag-NWs) nanocomposite.

First, the sandblasted Nafion membrane was dipped in the Pt complex solution for over 12 h. The Nafion membrane was placed in a basic solution bath at 40 °C, and 1.7 mL of the 5 wt% NaBH_4_ solution was added every 30 min seven times. During the process, the temperature was gradually raised to 60 °C. Next, 17 mL of the reducing agent was added for 1.5 h at 60 °C; the membrane was rinsed with a basic solution and immersed in dilute 0.1 N HCl for 1 h. After this process was repeated more than 4 times, the Nafion membrane was placed in a Pt-complex bath at 40 °C and 5 mL of the 5 wt% NH_2_OH solution was added. Then, 6 mL of the hydroxylamine hydrochloride solution and 3 mL of the hydrazine solution were added every 30 min eight times. During this process, the temperature gradually increased to 60 °C. Finally, the membrane was immersed in dilute 0.1 N HCl for 30 min.

The fabricated actuator was immersed in the LiCl solution for over 12 h for ion exchange to take place. After that, the actuator was placed between filter papers, inserted in a vacuum oven, pressed to a pressure of 35.28 mPa, and kept in the oven at 110 °C for over 12 h. The Nafion membrane was immersed in the IL solution and kept in it for 4.5 h at 150 °C for ion substitution to take place. Finally, the actuator was placed between filter papers, inserted in the vacuum oven, pressed to a pressure of 35.28 mPa, and kept in the oven at 110 °C for 1 h. 

### 2.3. Fabrication of IEAP Actuator Based on PEDOT:PSS/(graphene–Ag-NWs) Nanocomposite

First, the Nafion membrane was immersed in the LiCl solution for over 12 h to allow ion exchange to take place, where the hydrogen ions (H^+^) from the ionic polymer in the actuator were exchanged with the alkali metal ions, i.e., lithium ions (Li^+^) from LiCl. After ion exchange, the actuator was placed between filter papers and placed in a vacuum oven, pressed to a pressure of 0.66 Pa, and kept in the oven for over 12 h at 120 °C. The Nafion was then immersed in the IL solution for 4.5 h at 150 °C to allow ion substitution to take place. After ion substitution, the actuator was placed between filter papers, placed in the vacuum oven, pressed to a pressure of 0.66 Pa, and kept in the oven for 1 h at 150 °C. This process injected moving cations in the Nafion membrane for subsequent actuator operation. 

**Figure 1:**
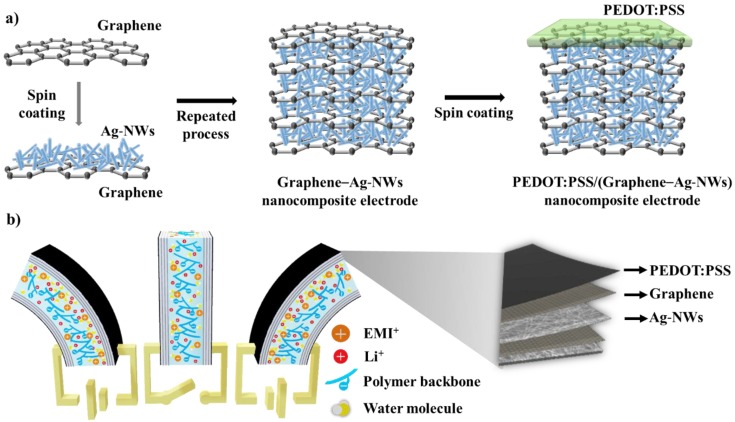
Schematic diagrams of IEAP actor based on PEDOT:PSS/(graphene–Ag-NWs) nanocomposite electrodes: (**a**) synthesis of a PEDOT:PSS/(graphene–Ag-NWs) nanocomposite electrode; (**b**) design of IEAP actuator based on PEDOT:PSS/(graphene–Ag-NWs) nanocomposite electrodes.

Figure 1a shows a schematic diagram of the fabrication of the PEDOT:PSS/(graphene–Ag-NWs) nanocomposite for the electrodes of the IEAP actuator. Next, the CVD-grown graphene and the catalytic Cu substrate (hereafter referred to as graphene/Cu) were prepared. A 5 wt% PMMA solution was spin-coated (4000 rpm, 30 s) on the graphene/Cu surface and dried (80 °C, 10 min). The back side of the PMMA/graphene/Cu system was then etched by 20% nitric acid for 3 min and rinsed with DI water. The Cu layer was etched away by a 0.1 M APS solution for 4 h. The remaining PMMA/graphene was rinsed with DI water and dried in the vacuum oven after the two layers were transferred onto the Nafion membrane. The PMMA layer was then removed by etching in acetone for 30 s. The Ag-NWs were spin-coated (500 rpm, 10 s) onto the graphene/Nafion surface and dried (80 °C, 30 min). This process was repeated more than four times to form the nanocomposite electrode layer. Finally, PEDOT:PSS was spin-coated (150 rpm, 50 s) onto the graphene surface and dried (100 °C, 2 min) to form the last layer of electrode. The same PEDOT:PSS/(graphene–Ag-NWs) nanocomposite electrode was also formed on the opposite side of the Nafion membrane using the same process. In order to improve the actuation performance of the IEAP actuator, the PEDOT:PSS/(graphene–Ag-NWs) nanocomposite was used as the electrodes, as shown in Figure 1b. These PEDOT:PSS/(graphene–Ag-NWs) nanocomposite-based IEAP actuators operate based on ion mobility and redistribution within their macromolecular network in an electrical field. These polymeric strips can operate in air or in water. They absorb water from air to keep them moist and enhance cation mobility within the molecular network. In our study, the IEAP actuator was operated at room temperature in air. An applied electric voltage affected the cation distribution within the membrane, forcing the cations to migrate towards the cathode. The voltage (potential difference) caused the ions to transfer in the cluster of ionic strips that provided the actuation. An IEAP actuator is also flexible because of its low stiffness, which produces a large bending deflection by applying a small voltage (1–5 V). 

### 2.4. Characterization

**Figure 2:**
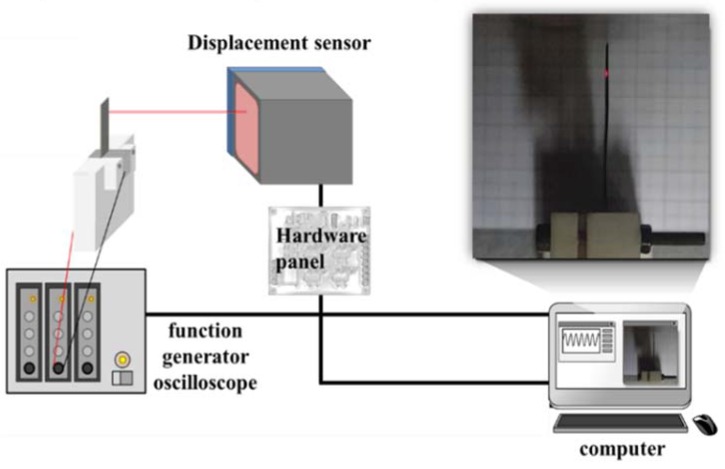
Experimental set-up used for measurements of IEAP actuator performance.

The morphology of the electrodes was investigated using field-emission scanning electron microscopy (FE-SEM; S-4300, Hitachi, Japan). X-ray diffraction (XRD; Cu Kα radiation, *λ* = 1.541874 Å) was used to investigate their structural properties. X-ray photoelectron (XP) spectroscopy (XPS) data were obtained using an XP spectrometer (ESCALAB 250, Thermo Fisher Scientific, Seoul, Korea). Our research group constructed an actuation performance analyzer to evaluate the actuator. A schematic representation of the analyzer is shown in Figure 2.

The electrical conductivity (*σ*) was calculated using the following equation:

(1) $$\sigma =\frac{1}{R_{s}\times t},$$

where *R*_s_ and *t* are the sheet resistance and thickness, respectively.

The water uptake potential (WUP) was calculated using the following equation:

(2) $$\mathrm{WUP}=\frac{W_{w}-W_{d}}{W_{d}}\times 100,$$

where $W_{w}$ is weight of the wet ionic polymer membrane, and $W_{d}$ is weight of the dry ionic polymer membrane.

The curvature (*κ*) was calculated as follows:

(3) $$\kappa =\frac{2\delta }{l^{2}\times \delta^{2}},$$

where δ and l are the tip displacement and the free length of the actuator, respectively.

## 3. Results

**Figure 3:**
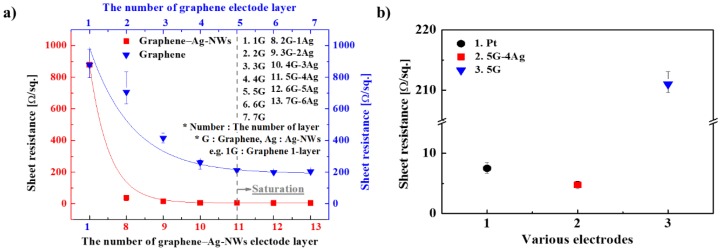
(**a**) Sheet resistance of graphene–Ag-NW composites and graphene according to the number of layers; (**b**) Sheet resistance Pt, 5G–4Ag, and 5G electrodes.

Graphene–Ag-NW nanocomposite electrodes were prepared for the IEAP actuator, and the entire surface of each electrode must have uniform sheet resistance. When the graphene and the graphene–Ag-NW nanocomposite were prepared layer-by-layer, as shown in Figure 1, the sheet resistance of the electrodes decreased linearly as the number of layers increased, as shown in Figure 3a. When the sheet resistance of the 5-layered graphene (5G) electrode reached saturation, it had a value of about 200 Ω/sq. When the sheet resistance of the nanocomposite electrode with 5 graphene layers and 4 Ag-NW layers (5G–4Ag) reached saturation, it had a value of about 4 Ω/sq. The standard deviation of the sheet resistance decreased as the sheet resistance decreased, indicating that the electrode had uniform sheet resistance over the entire surface. Figure 3b shows the sheet resistance of a conventional Pt electrode, the 5G electrode, and the 5G–4Ag electrode, indicating that the 5G–4Ag electrode had lower sheet resistance than the Pt and 5G electrodes.

**Figure 4:**
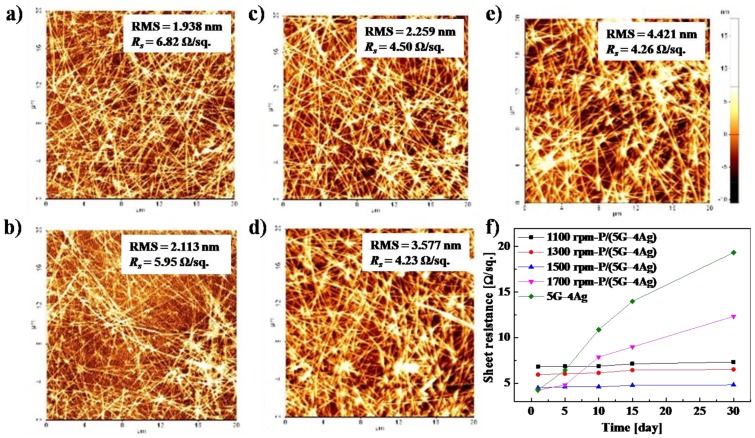
AFM images of the PEDOT:PSS-coated 5G–4Ag electrode prepared at different spinning speeds: (**a**) 1100 rpm; (**b**) 1300 rpm; (**c**) 1500 rpm; (**d**) 1700 rpm; (**e**) Uncoated 5G–4Ag electrode; the inset shows the RMS roughness and *R*_s_ of each sample; (**f**) Changes in sheet resistance during a period of 30 days in a natural environment.

To smoothen the surface and increase the durability of the electrode, we spin-coated PEDOT:PSS onto the 5G–4Ag electrode at different spinning speeds. Figure 4a–e shows the root-mean-square (RMS) roughness and sheet resistance of the 5G–4Ag electrode with coatings prepared at spinning speeds from 1100 to 1700 rpm. As the spinning speed increased, the RMS roughness of the PEDOT: PSS-coated 5G–4Ag electrode changed from 4.421 to 1.938, 2.113, 2.259, and 3.577 nm, and the sheet resistance changed from 4.26 to 6.82, 5.95, 4.50, and 4.23 Ω/sq. The RMS roughness of the 5G–4Ag electrode with PEDOT: PSS spin-coated at 1500 rpm was significantly reduced, and it did not have a negative effect on the sheet resistance. 

Moreover, Figure 4f shows the changes in sheet resistance of the PEDOT:PSS-coated 5G-4Ag electrode prepared at different spinning speeds during a period of 30 days in a natural environment. The sheet resistance of the 5G–4Ag electrode with PEDOT:PSS spin-coated at 1500 rpm was not increased, as shown in Figure 4f, despite being exposed to air for 30 days. These results show that the rough surface and durability of the 5G–4Ag electrode improved when the PEDOT:PSS layer was spin-coated at 1500 rpm. Therefore, we fabricated the IEAP actuator with an electrode based on 5G–4Ag with a PEDOT:PSS coating prepared at 1500 rpm (P/(5G–4Ag)), and it was expected to exhibit fast and stable actuation.

### 3.1. Characterization of the PEDOT:PSS/(graphene–Ag-NWs) Nanocomposite Electrode

**Figure 5:**
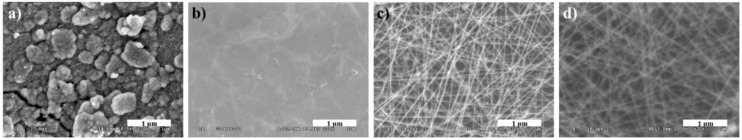
SEM images of fabricated electrodes: (**a**) Pt; (**b**) 5G; (**c**) 5G-4Ag; (**d**) P/(5G–4Ag).

Figure 5 shows the surface of the fabricated Pt, 5G, 5G–4Ag, P/(5G–4Ag) electrodes. The surface of the Pt electrode was very rough, but the surface of the 5G, 5G-4Ag, and P/(5G-4Ag) electrodes were smooth. The thickness of each electrodes was also measured. 

The basic parameters, such as thickness, sheet resistance, and electrical conductivity, of the electrodes of various types are summarized in Table 1. The P/(5G–4Ag) nanocomposite electrode had the lowest sheet resistance (*R*_s_: ~4.5 Ω/sq.) among the electrodes. It had superior electrical conductivity that was about 259 times that of the Pt electrode and about 12 times that of the 5-layered graphene electrode. 

Satisfactory sheet resistance and electrical conductivity of electrodes used in ionic polymer actuators are key factors of high-performance actuation because interfacial or contact resistance between the electrodes and polymer membranes highly affect the electric field in the actuator and the performance of the actuator. Compared to the other electrodes, the P/(5G–4Ag) nanocomposite electrode is the most suitable choice for an ionic polymer actuator because of its low sheet resistance, high conductivity, and high flexibility for larger bending deformations.

**Figure 6:**
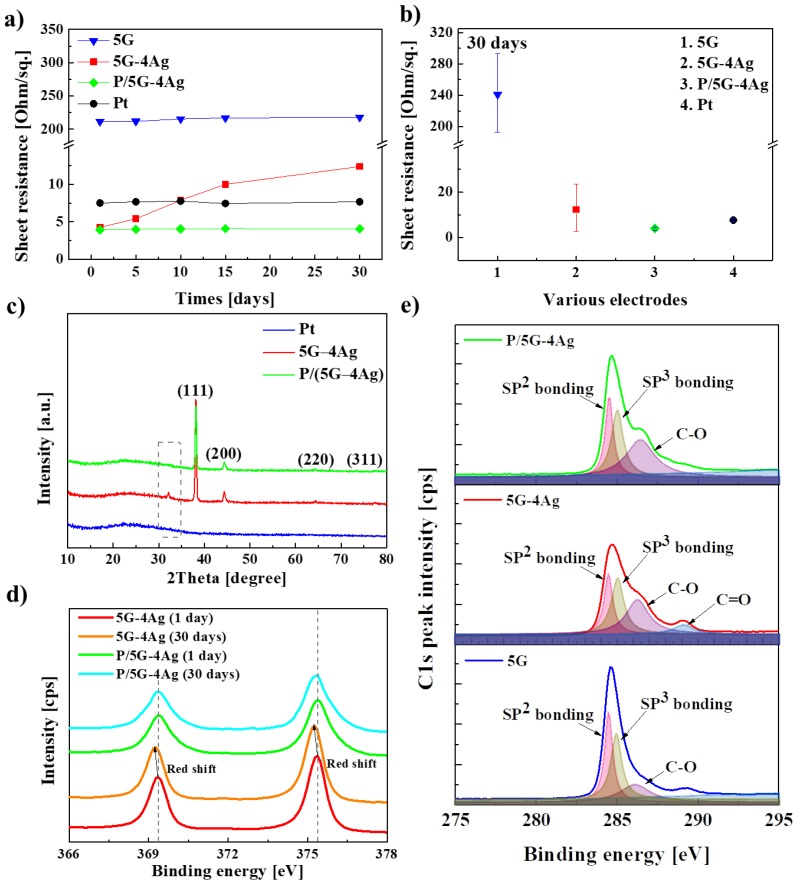
(**a**) Sheet resistance of electrodes as a function of time; (**b**) Sheet resistance of electrodes after exposure to the natural environment for 30 days; (**c**) XRD spectra of electrodes; XPS spectra showing (**d**) Ag 3d peaks according to the binding energy of the electrodes and (**e**) C 1s peak according to the binding energy of the electrodes.

To apply an actuator to the field biomimetics, its electrode must be resistant to various elements in the natural environment. Therefore, the durability and degree of oxidation of the electrode must be considered. The electrodes fabricated in our study were exposed to the natural environment for about 5, 10, 15, and 30 days. Figure 6a shows the sheet resistance of the 5G, 5G–4Ag, P/(5G–4Ag), and Pt electrodes during their exposure to the natural environment for 30 days. The electric conductivity of P/(5G–4Ag), Pt, and 5G electrodes remained unchanged, and the electrical conductivity of the 5G–4Ag electrode increased over time. After 30 days, the P/(5G–4Ag) nanocomposite electrode also had the lowest sheet resistance among the electrodes (Figure 6b).

The chemical characteristics of the 5G, 5G–4Ag, and P/(5G–4Ag) after exposure to the natural environment for 30 days were studied using XRD and XPS. Figure 6c shows a broad peak around 25° in the XRD pattern of 5G, which can be attributed to the small size of the layers or a relatively short-range domain order in the stacked sheets [44,45,46].The peaks around 38.1°, 44.4°, 64.4°, and 77.3° in the XRD pattern of 5G–4Ag and P/(5G–4Ag) correspond to the (111), (200), (220), and (311) reflection planes of face-centered cubic Ag [44,45,46], but a small peak at 32.8°, corresponding to Ag_2_O [44], was observed in the XRD pattern of 5G–4Ag. The formation of Ag_2_O was due to the oxidation of Ag-NWs in the natural environment. However, the small peak is absent in the XRD pattern of PEDOT:PSS-coated 5G–4Ag, indicating that PEDOT:PSS acted as an encapsulation layer that protected the Ag-NWs from surface oxidation, thus improving the chemical stability of the hybrid. 

Figure 6d shows the XPS spectra about the Ag 3d peak of various electrodes [44]. In general, the Ag 3d peak can be separated to Ag 3d_3/2_ peak and Ag 3d_5/2_ peak. The Ag 3d_3/2_ peak is caused by down-spin electrons in the d-orbital in the M-shell of silver, while the Ag 3d_5/2_ peak is caused by up-spin electrons at the same position as those related to Ag 3d_3/2_. The XPS spectra of the electrodes, except for the 5G electrode, all exhibit the Ag 3d_3/2_ peak and Ag 3d_5/2_ peak at 369 and 375 eV, respectively. The binding energy associated with each Ag 3d peak is slightly shifted (by ~0.2 eV) toward lower values for uncoated electrode. Generally speaking, Ag_2_O and AgO have binding energies that are 0.3–0.6 eV lower than Ag owing to the lowered binding energy when oxidation of Ag occurs. Therefore, the shift of the peak towards lower value indicates the formation of silver oxide. Figure 6e shows the XP spectra of the C 1s peak according to the binding energy of the electrodes. The synthesized graphene exhibited a complex C 1s spectrum containing contributions from sp^2^ and sp^3^ bonding. The sp^3^ carbon peak is positioned 1.0 eV away toward the higher binding energy side) from the sp^2^ peak. There were more sp^2^ carbon than sp^3^ carbon caused by C–C bonding in the CVD-grown graphene, and a C–O bonding peak was observed. The sp^2^ and sp^3^ bonding peaks have lower intensity in the spectrum of the uncoated nanocomposite than in the spectrum of the 5-layered graphene. C–O and C=O bonding peaks were also observed. However, the sp^2^ and sp^3^ bonding peaks of in the spectrum of the PEDOT:PSS-coated nanocomposite have higher intensity, and the intensity of the C–O bonding peak remains unchanged owing to the PEDOT:PSS coating with C–C and C–O bonding. Moreover, the peak caused by C=O bonding was not observed in the spectrum of the uncoated 5G–4Ag nanocomposite. Consequently, as shown in Figure 6, when the electrodes were exposed to the outside for a long time, the increase in sheet resistance was caused by the oxidation of carbon and silver in 5G and 5G–4Ag. In addition, we found that the oxidation of an electrode could be improved through the PEDOT:PSS coating.

### 3.2. Actuation Performance of IEAP Actuator Based on PEDOT:PSS/(graphene–Ag NWs) Nanocomposite

**Figure 7:**
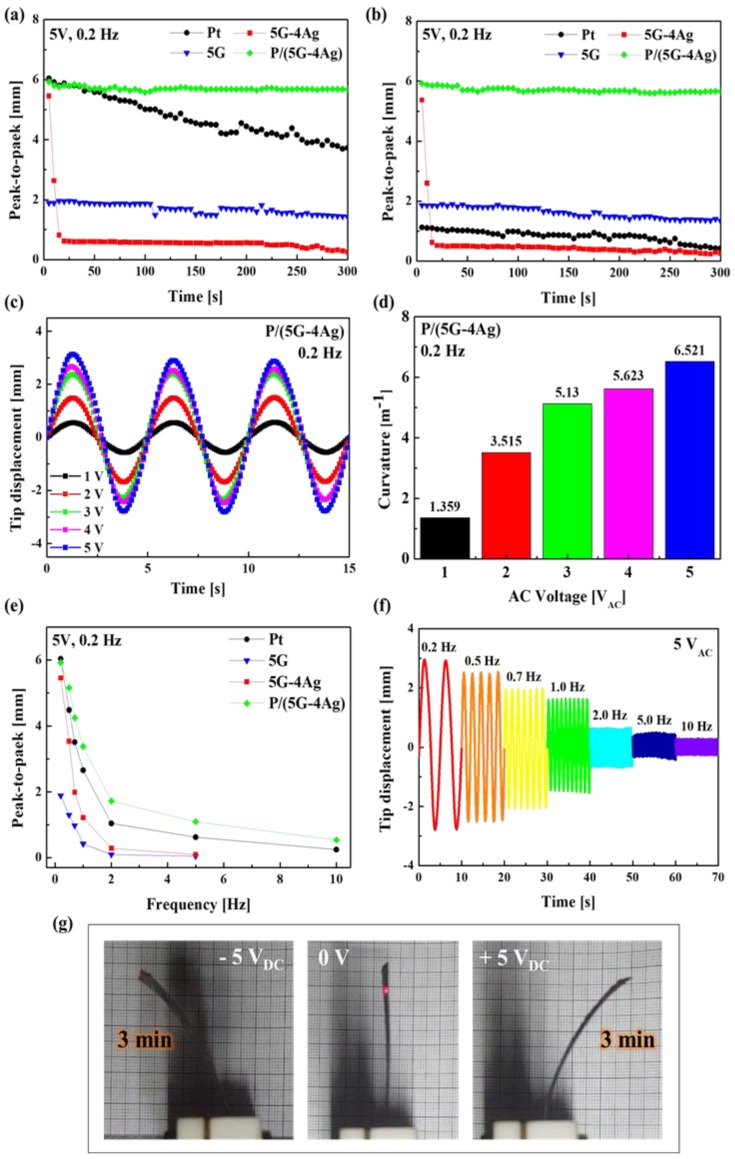
Open-air actuation performances of fabricated electroactive polymer actuators based on Pt, 5Gl, 5G–4Ag, P/(5G–4Ag) electrodes (**a**) before and (**b**) after drying. Bending actuation performance of the P/(5G–4Ag) actuator (**c**) under various input voltages and (**d**) their corresponding curvatures. (**e**) Bending displacement of different actuators at different frequencies. (**f**) Bending displacement of the P/(5G–4Ag) actuator at different frequencies. (**g**) The bending motion photograph of P/(5G–4Ag) actuator under the input voltage of 5 V_DC_.

We measured and observed the actuation performance of IEAP actuators with different electrodes. The actuation performances of the fabricated electroactive polymer actuators were evaluated, and the results are presented in Figure 7. In order to investigate the effect of electrode type on each actuator, the actuation performances of Nafion-based actuators with four kinds of electrode (Pt, 5G, 5G–4Ag, P/(5G–4Ag)) were measured under sinusoidal wave inputs with a peak voltage of 5 V and excitation frequency of 0.2 Hz, as shown in Figure 7a,b. 

The results of the durability test are shown in Figure 7a; the actuator with the P/(5G–4Ag) electrode showed a slight degradation (within 5%) of the tip displacement during 300 s, while the actuators with 5G, Pt, and 5G–4Ag electrodes showed dramatic degradation below 25%, 61%, and 96%, respectively, as time went on during the 300 s period. The performance of the 5G actuator was relatively stable, but its displacement characteristics were very poor when compared to those of other electrodes. The performance degradation of the Pt actuator resulted from the leakage of evaporated water molecules via the cracked surfaces of the platinum electrodes. The performance degradation of the 5G–4Ag actuator resulted from peeling of the electrode surface because of the high contact resistance and heat due to the irregular surface, and the poor adhesion of Ag-NWs. Water molecules in the graphene-based actuator did not evaporate because of the smooth surface morphology, hydrophobicity, and low permeability of the graphene-based electrode. To more clearly demonstrate this, we dried the actuators in an oven at 110 °C for 48 h. Next, as shown in Figure 7b, we compared the actuation performances of four actuators after the drying process. The actuation performances after the drying process were associated with the water-impermeable properties that could prevent the leakage of inner water molecules, even under extreme drying conditions. When the actuation performances of the four actuators were observed during 300 s under electric stimulation, the P/(5G–4Ag), 5G–4Ag, and 5G actuators exhibited actuation behavior similar to that before drying, as shown in Figure 7a. In contrast, the actuation behavior of the Pt-based actuator (maximum peak-to-peak distance: 1.12 mm) was found to be less robust than the actuation behavior (maximum peak-to-peak distance: 6.04 mm) before drying, as shown in Figure 7a.

The total weight of the Pt, 5G, 5G–4Ag, and P/(5G–4Ag) actuators before and after drying were measured to check the weight loss resulting from the evaporation of water; the results are listed in Table 2. Also, using Equation (2), the water uptake potential (WUP) was calculated for each actuator, and the results are also shown in Table 2. In addition, using Equation (2), the water uptake potential (WUP) was calculated for each actuator, and the results are shown in Table 2. As its name suggests, the WUP was used to determine the water-absorbing capacity of the actuators with the prepared electrodes. All actuators, except the one with Pt electrodes (i.e., actuators with 5G, 5G–4Ag, and P/5G–4Ag electrodes), had a WUP of around 20%. On the other hand, the actuator with Pt electrodes had a WUP of only ~14% because the carbon and allied allotropes had the capacity for water absorption and water retention. The weight loss exhibited by the 5G, 5G–4Ag, and P/5G–4Ag actuators was under 5% after drying, while the weight loss of the Pt actuator was ~20% owing to the leakage of water molecules through the cracked metallic electrode during the drying process. The water-absorbing performance and weight loss may affect the durability of the carbon-based actuators.

Figure 7c shows the harmonic responses of the P/5G–4Ag actuators under five different driving voltages (1–5 V) at an excitation frequency of 0.2 Hz. As the driving voltage increased, the tip displacement of the P/(5G–4Ag) actuator increased. The maximum tip displacement of the P/(5G–4Ag) actuator reached 0.55, 1.49, 2.37, 2.67, and 3.13 mm under input voltages of 1, 2, 3, 4, and 5 V, respectively. By using Equation (3), the bending curvatures (*κ*) were calculated as shown in Figure 7d. We obtained *κ* = 1.359, 3.515, 5.130, 5.623, and 6.521 m^−1^ under the input voltages of 1, 2, 3, 4, and 5 V, respectively. Moreover, the bending performances of all actuators were measured under an input voltage of 5 V_AC_ at various frequencies from 0.2 to 10 Hz, and the results are shown in Figure 7e. The P/(5G–4Ag) actuator showed the best actuation performance compared with other actuators at various frequencies, and it showed stable actuation performance at high frequency (Figure 7f). The photograph of Figure 7g shows images of the bending P/(5G–4Ag) actuator under the input voltage of 5 V_DC_ after 3 min (instantaneous time). Therefore, these results demonstrate that the application of the P/(5G–4Ag) nanocomposite to both electrodes greatly improved the overall actuation performances (such as the fast response rate, durability and stability) of soft ionic actuators.

## 4. Discussion and Conclusions

In summary, we developed a P/(5G–4Ag) nanocomposite electrode with a very low sheet resistance of ~4 Ω/sq. It also had higher electrical conductivity and a smoother surface (without cracks) than a conventional Pt electrode. The Ag-NWs contributed to the increased electrical conductivity of graphene electrode. The PEDOT:PSS coating provided sufficient protection to prevent oxidation of the electrode, and the strong adhesion between the ionic polymer and electrode ensured that the electrical conductivity was not reduced. A fast and stable IEAP artificial muscle with superior large bending deformations was developed based on the P/(5G–4Ag) nanocomposite that provided an efficient pathway for electron and ion transport. The durability problem, which was the main drawback of conventional Pt actuators, was resolved by utilizing liquid-impermeable, hydrophobic, highly flexible, and conductive P/(5G–4Ag) electrodes. Unlike the metallic electrodes in conventional IPMC actuators that are deposited by electroless plating in, the P/(5G–4Ag) electrode had a smooth outer surface without apparent cracks in the direction of increasing thickness, and it was nearly liquid-impermeable. The results of the durability test for the actuator with P/(5G–4Ag) electrodes showed a slight degradation (within 5%) of the tip displacement during a period of 300 s, while the actuators with 5G, Pt, and 5G–4Ag electrodes showed dramatic degradation below 25%, 61%, and 96%, respectively, with increasing time during the 300 s period. Moreover, the P/(5G–4Ag) actuator showed the best actuation performance at various frequencies and exhibited stable actuation at high frequency (~10 Hz). The newly designed P/(5G–4Ag) electrode, which had the unique ability to prevent leakage of the vaporized or liquid electrolyte and mobile ions during excitation by electrical stimuli, is expected to greatly contribute to an exceptionally durable, fast and stable IEAP actuator. The proposed IEAP actuator based on the P/(5G–4Ag) nanocomposite electrode can be used in active biomedical devices, biomimetic robots, wearable electronics, and flexible soft electronics.

## Figures and Tables

**Table 1:** Thickness, sheet resistance and electrical conductivity of various types of electrode.

Electrode Types	Thickness, *t* [nm]	Sheet Resistance, *R*_s_ [Ω/sq.]	Electrical Conductivity, *σ* [S/m]
Pure graphene [38,39,40,41]	Monolayer	30	~ 10^8^
CVD-grown graphene [40]	Four layers/ monolayer	350/2100	-
CVD-grown graphene on Ni [42]	6–10 layers	280	-
Reduced-graphene-oxide paper [9,43]	10 × 10^3^/5 × 10^3^	13.89/6.35	3.15 × 10^2^
Reduced-graphene-oxide flake [44]	1.00	5 × 10^6^	-
Pt [this work]	16.92 × 10^3^	7.50	78.8 × 10^2^
5G [this work]	15.69	211.75	30.10 × 10^4^
5G–4Ag [this work]	112.22	4.23	21.07 × 10^5^
P/(5G–4Ag) [this work]	122.25	4.50	18.18 × 10^5^

**Table 2:** Weight change and water uptake potential of Pt, 5G, 5G–4Ag, and P/(5G–4Ag) actuators.

Electrode Type	Weight [g]			WUP [%]	Weigh Loss [%]
	Before Water Absorption (*W*_d_)	After Water Absorption (*W*_w_)	After Drying		
**Pt**	0.112	0.128	0.102	14.28%	20.31%
**5G**	0.031	0.037	0.035	19.35%	5.40%
**5G–4Ag**	0.043	0.052	0.050	20.93%	3.85%
**P**/(**5G–4Ag**)	0.050	0.061	0.060	22.00%	1.64%
